# A Synaptic Framework for the Persistence of Memory Engrams

**DOI:** 10.3389/fnsyn.2021.661476

**Published:** 2021-03-24

**Authors:** Priyanka Rao-Ruiz, Esther Visser, Miodrag Mitrić, August B. Smit, Michel C. van den Oever

**Affiliations:** Department of Molecular and Cellular Neurobiology, Center for Neurogenomics and Cognitive Research, Amsterdam Neuroscience, Vrije Universiteit Amsterdam, Amsterdam, Netherlands

**Keywords:** memory engram, engram cell network, synaptic connectivity, memory persistence, cellular feedback loops

## Abstract

The ability to store and retrieve learned information over prolonged periods of time is an essential and intriguing property of the brain. Insight into the neurobiological mechanisms that underlie memory consolidation is of utmost importance for our understanding of memory persistence and how this is affected in memory disorders. Recent evidence indicates that a given memory is encoded by sparsely distributed neurons that become highly activated during learning, so-called engram cells. Research by us and others confirms the persistent nature of cortical engram cells by showing that these neurons are required for memory expression up to at least 1 month after they were activated during learning. Strengthened synaptic connectivity between engram cells is thought to ensure reactivation of the engram cell network during retrieval. However, given the continuous integration of new information into existing neuronal circuits and the relatively rapid turnover rate of synaptic proteins, it is unclear whether a lasting learning-induced increase in synaptic connectivity is mediated by stable synapses or by continuous dynamic turnover of synapses of the engram cell network. Here, we first discuss evidence for the persistence of engram cells and memory-relevant adaptations in synaptic plasticity, and then propose models of synaptic adaptations and molecular mechanisms that may support memory persistence through the maintenance of enhanced synaptic connectivity within an engram cell network.

## Introduction

Our memories define who we are, help us make decisions and guide our behavior. The ability to effectively encode, store and retrieve information is therefore an essential feature of life. Although the recollection of most experiences fades with time, certain memories are retained for many years or even a lifetime. How the brain is able to process and persistently store learned information has been a topic of intense research for a long time and great progress has been made in recent years toward a better understanding of the mechanisms underlying memory persistence.

Memory formation is initiated by the integration of external and interoceptive sensory stimuli in neuronal circuits, forming a cohesive representation of a specific event. Subsequently, the neurons involved are thought to undergo physical changes that enable retrieval of the learned information. The physical representation of experience-driven changes in the brain is collectively referred to as a memory engram (Box 1), a term that gained popularity in recent years (Josselyn et al., 2015), but that was first introduced by the German scientist Richard Semon in the early 20th century (Semon, 1911). Learning-induced changes do not occur globally or randomly within memory-relevant brain regions. Instead, accumulating evidence indicates that sparse ensembles of neurons become highly activated during learning and act as a substrate for the storage of a memory engram (Whitaker and Hope, 2018; Josselyn and Tonegawa, 2020). As such, the neurons that encode a memory through consolidation of learning-induced physical adaptations (i.e., the engram) are called engram cells (Tonegawa et al., 2015).

**Box 1. Key definitions.**

**Memory engram:** the physical representation of learning-induced changes in the brain that are causal to memory storage and retrieval.

**Engram cells:** neurons that encode a memory through consolidation of learning-induced physical adaptations. These neurons are highly activated during learning and subsequently act as a substrate for the storage of a memory engram.

**Engram cell network:** a network of engram cells that persists through the establishment of engram synapses.

**Engram synapses:** synapses between engram cells that are formed or strengthened as a result of learning to ensure reactivation of the engram cell network during memory retrieval.

**Synaptic connectivity:** collection of structural (size and number) and electrophysiological (strength and efficacy) properties of synapses. Learning augments one or more of these properties to enhance synaptic connectivity between engram cells.

**Global synaptic adaptations:** learning-induced changes in synaptic connectivity that have been measured in a random population of neurons in a given brain region, without discriminating between engram and non-engram cells.

**Engram cell-specific synaptic adaptations:** learning-induced changes in synaptic connectivity that have been measured selectively in engram cells in a given brain region, often by comparison with non-engram cells.

**Cellular feedback loops:** recurrent cause-and-effect sequences where the output of an intracellular molecular pathway influences its input.

**FIGURE 1:**
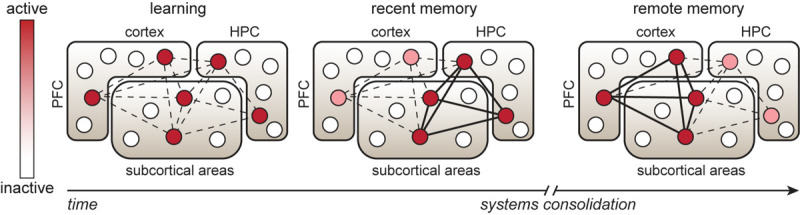
Temporal reorganization of an engram cell network. Learning is associated with activation of neuronal ensembles (indicated by circles) in memory-relevant brain regions, including hippocampal, cortical and subcortical areas. These specific neurons consolidate the physical changes that support memory storage and retrieval and are therefore considered engram cells. During the first post-learning hours to days, strengthening of synaptic connectivity occurs between engram cells in the hippocampus (HPC) and other areas to enable recent memory expression. Over the following days to weeks, systems consolidation, involving replay and synchronization of learning-associated neuronal activity patterns in HPC and cortical regions, such as the prefrontal cortex (PFC), is thought to promote maturation of cortical engram cells. This process drives strengthening of synaptic connectivity between cortical-cortical and cortical-subcortical engram cells and a progressive shift in the engram cell network toward a higher dependency on cortical engram cells for remote memory expression.

The initial process of long-term memory formation through cellular consolidation of experience-driven changes occurs on a time-scale of hours to days and requires *de novo* protein synthesis (McGaugh, 2000). However, for a memory to persist and to be retrieved at remote timepoints (>2 weeks) after learning, it is thought to undergo a process of systems consolidation (Frankland and Bontempi, 2005), which involves temporal reorganization of the cells involved in the brain-wide engram network (Figure 1) (Tonegawa et al., 2018). More specifically, memory retrieval at recent timepoints (within the 1st week) after learning relies heavily on processing of learned information and consolidation of the engram in subregions of the hippocampus, as well as the amygdala and other subcortical structures (depending on the type of information that is processed), whereas systems consolidation drives the engagement of neocortical regions in memory retrieval (Takashima et al., 2006; Wheeler et al., 2013). In particular, areas of the prefrontal cortex are not involved in recent memory expression, but are required for adequate memory retrieval at remote timepoints (Frankland et al., 2004; Goshen et al., 2011; Lesburgueres et al., 2011). In line with this, expression of recent memories relies on engram cells in the hippocampus (Liu et al., 2012; Denny et al., 2014; Tanaka et al., 2014; Rao-Ruiz et al., 2019), whereas remote memories depend on reactivation of learning-activated neurons in areas of the prefrontal cortex (Kitamura et al., 2017; Matos et al., 2019), among other regions (Tayler et al., 2013). The progressive dependency on engram cells in neocortical regions is likely driven by post-learning synchronization and replay of neuronal activity in hippocampal and cortical areas (Squire et al., 2015; Xia et al., 2017). Interestingly, whereas prolonged optogenetic inhibition of the hippocampal CA1 region does not affect expression of remote contextual fear memory, temporally precise inhibition during a memory test does impair recall and reduces the increase in activity in the anterior cingulate cortex (ACC) normally observed at this timepoint (Goshen et al., 2011). This indicates that systems consolidation drives the necessity of cortical regions in remote memory, but interplay between the hippocampus and neocortex remains important for adequate remote memory retrieval under normal conditions. Notably, systems consolidation has been demonstrated for contextual memories (Frankland et al., 2004; Goshen et al., 2011), but also occurs after other types of learning, such as spatial learning (Teixeira et al., 2006) and social transmission of food preference (Lesburgueres et al., 2011).

The enduring neurobiological adaptations that ensure memory storage and persistent reactivation of engram cells during memory retrieval are largely unknown. At the level of neuronal physiology, changes in excitability and synaptic plasticity have been observed in engram cells in various brain regions (Whitaker and Hope, 2018; Josselyn and Tonegawa, 2020). Alterations in intrinsic physiological properties of a neuron (i.e., membrane conductance) control the probability of action potential generation (Hille, 1978), thereby favoring or limiting synaptic vesicle release. As such, the excitability state of a neuron influences neurotransmission and contributes to synaptic changes involved in memory encoding and consolidation (Zhang and Linden, 2003; Chen L. et al., 2020). Several studies have demonstrated that the excitability state of a neuron affects its probability to be allocated in an engram network (Zhou et al., 2009; Yiu et al., 2014; Brebner et al., 2020). Furthermore, a transient learning-induced increase in excitability of engram cells is thought to mediate the linkage of experiences that occur close in time through co-allocation of engram cells (Cai et al., 2016; Rashid et al., 2016), whereas transient increased excitability that lasts up to 1 h after retrieval regulates the accuracy of subsequent memory retrieval (Pignatelli et al., 2019). Hence, changes in the excitability state of an engram cell have a critical role in memory processing. However, as most excitability changes that have been observed thus far are acutely induced by learning or memory retrieval, it is unclear how long-lasting these changes are and whether this underlies the retention of an engram cell network. Moreover, as a single neuron might participate in multiple engram cell networks, a persistent change in whole cell excitability would influence the processing of multiple memory engrams. Therefore, the persistence and reactivation of engram cell networks more likely depends on the strengthening of specific synaptic connections between neurons that fire together (Hebb, 1949). If learning modulates the properties of a subset of synapses on an engram cell (i.e., the engram synapses), that specific neuron would be able to contribute to other engram cell networks via its remaining synaptic connections, allowing for much greater flexibility and memory capacity in neuronal circuits. Hence, for the purpose of this review, we will focus predominantly on mechanisms of synaptic connectivity underlying the stability and reactivation of engram cell networks over time.

We will first summarize evidence of the existence of sparse and persistent engram cells. Secondly, we will provide an overview of learning-induced global and engram cell-specific synaptic changes associated with progressive memory stages. Finally, we will discuss synaptic models and molecular mechanisms that aim to explain how enhanced synaptic connectivity of the engram cell network is maintained over time to support memory persistence.

## Engram Cells Supporting Memory Persistence

The concept that memories are encoded by synchronized firing of neuronal ensembles was first postulated by Hebb (1949). This theory was initially supported by studies that used *in vivo* electrophysiological recordings as a measure of neuronal activity or the expression of immediate early genes (IEGs) as a proxy thereof (John and Schwartz, 1978; Sheng and Greenberg, 1990). Recent technological advances enabled researchers to causally link neuronal ensemble activity with the expression of learned behavior. These approaches are based on engineered viral vectors and/or transgenic rodent lines that either bias the allocation of neurons into an engram cell network or that make use of the transcriptional promoter of an IEG, such as *Fos* or *Arc*, to drive expression of a transgene in neurons that are activated during an experimenter-defined time-window (Cruz et al., 2013; Josselyn et al., 2015). When combined with targeted cell ablation, opto- or chemogenetics, this enables selective manipulation of neuronal ensembles and thereby the analysis of gain- or loss-of-function.

Aversive and appetitive learning paradigms in rodents have been used to determine whether neuronal ensemble activity causally supports memory expression. In particular, learning-activated neuronal ensembles that control expression of recently acquired aversive and appetitive memories have been identified in the amygdala (Han et al., 2009; Zhou et al., 2009; Hsiang et al., 2014; Redondo et al., 2014; Gore et al., 2015), hippocampus (Liu et al., 2012; Denny et al., 2014; Tanaka et al., 2014; Rao-Ruiz et al., 2019) and retrosplenial cortex (Cowansage et al., 2014) among other regions. With respect to appetitive memories, several studies have shown that neurons in the striatum and prefrontal cortex that are activated during expression of conditioned responses, such as cue-induced food or drug seeking and context-dependent locomotor sensitization, are required for subsequent expression of the same behavior (Koya et al., 2009; Bossert et al., 2011; Cruz et al., 2014; Suto et al., 2016; Warren et al., 2016; Caprioli et al., 2017; Wall et al., 2019). These findings, among others, have provided invaluable insight into the causal relationship between neuronal ensemble activity and conditioned behavior. As such, the neurons that are highly activated during learning and subsequently necessary for memory expression are thought to harbor the engram. However, in most early studies, the interval between the behavioral tagging/ablation session and the session during which the effect of the ensemble manipulation was determined was typically ∼1–7 days, which left the question of whether activated neuronal ensembles function as engram cells responsible for the persistence of a specific memory unresolved.

To enable visualization and manipulation of neuronal ensembles over prolonged periods of time, it is necessary to induce a stable or permanent tag in activated neurons. Tayler et al. (2013) used a TetTag transgenic mouse line to express a stable form of GFP in neurons that were activated during contextual fear conditioning (CFC). They observed context-dependent reactivation of tagged neurons in hippocampal and cortical regions 2 days after conditioning, whereas reactivation selectively occurred in several cortical regions upon retrieval 2 weeks later. More recently, Kitamura et al. (2017) combined the TetTag mouse with optogenetic manipulation and reported that CFC-activated neurons in the medial prefrontal cortex (mPFC) are sufficient and necessary for memory expression 12–14 days after conditioning. In line with this, we found, using a viral-TRAP (Targeted Recombination in Active Populations) combined with chemogenetics approach, that CFC-activated mPFC neurons are required for remote memory expression up to at least 1 month after learning (Matos et al., 2019). It is noteworthy that in both studies, mPFC engram cells were not involved in recent memory expression, supporting their time-dependent engagement through systems consolidation. The temporal role of mPFC engram cells was further demonstrated in a study that tagged these cells during memory retrieval at different timepoints after fear conditioning using a TRAP2 transgenic mouse (DeNardo et al., 2019). Interestingly, the involvement of CFC-activated mPFC neurons in remote memory appears to be contingent upon training intensity, with strong fear conditioning resulting in a disengagement of the learning-activated mPFC ensemble (Matos et al., 2019). We speculate that the encoding of highly aversive experiences depends on evolutionary more primordial emotional brain systems, consequently leading to a lack of top-down control by the mPFC. The persistent nature of memory encoding by mPFC neurons was also demonstrated in a mouse alcohol self-administration paradigm (Visser et al., 2020). We found that mPFC neurons that are activated during alcohol self-administration drive cue-induced relapse to alcohol seeking following 1 month of abstinence. Thus, even though investigations into engram cell stability gained attention only recently, it is evident that the mPFC can serve as a critical network hub, harboring persistent engram cells that encode aversive and appetitive types of memory.

## Learning-Associated Adaptations in Synaptic Connectivity

Augmentation of synaptic connectivity through changes in the structural and/or electrophysiological properties of synapses is considered to be crucial for memory consolidation, storage and retrieval. Collectively, these adaptations may ensure enhanced synaptic connectivity between engram cells and efficient reactivation of the network upon exposure to reminder cues. At an experimental level, several criteria need to be met to link synaptic connectivity with the stabilization of memories (Martin et al., 2000). These include evidence that (1) learning induces the formation of new connections and/or the structural remodeling of pre-existing ones, (2) learning induces detectable changes in network-specific synaptic physiology, (3) mimicking or manipulating altered synaptic connectivity (via criterium 1 and/or 2) installs or disrupts memory, respectively. In the following sections, we will highlight several key observations that fulfill one or more of these criteria.

### Global Synaptic Adaptations Associated With Recent Memory

In line with the aforementioned criteria, numerous studies have provided evidence that enhanced synaptic connectivity is associated with the formation and retrieval of long-term memories. For instance, studies using Golgi impregnation and electron microscopy demonstrated that associative learning enhances spine density and the formation of multiple-synapse boutons on hippocampal CA1 neurons (Geinisman et al., 2001; Leuner et al., 2003) and enlarges the postsynaptic density of spines in the lateral amygdala (Lamprecht et al., 2006). Moreover, *in vivo* transcranial two-photon imaging has shown that fear learning and subsequent extinction learning results in elimination and formation, respectively, of spines on the same dendritic branches in the frontal association cortex of mice (Lai et al., 2012). Lai et al. (2012) also elegantly demonstrated that the degree of structural plasticity correlated with behavioral responses of the animal and that reconditioning eliminates spines that were formed after extinction learning. The latter structural changes argue against the concept that learning enhances synaptic connectivity, however, whether the strength of remaining synapses was altered as a result of learning was not examined. Other studies suggest that learning-induced enhancement of synaptic connectivity is localized to ‘dendritic hotspots’, in clusters of synapses (Govindarajan et al., 2006; Frank et al., 2018). These clusters, rather than single synaptic contacts, could function as the fundamental unit of information storage. For instance, at a computational level, biophysical modeling suggests such clustered plasticity supports sparsity and increases memory capacity of neuronal systems. This was supported by two-photon imaging of the retrosplenial cortex in Thy1-YFP-H mice, which demonstrated that while contextual fear conditioning does not alter the overall rate of spine turnover, it results in clustered spine formation causally related to learning over 5 days of training (Frank et al., 2018). Thus, learning induces the formation of new, potentially clustered connections and the remodeling of existing connections and these structural changes in turn can support augmented synaptic connectivity patterns that subserve long-lasting memory storage.

Evidence for synaptic potentiation is based on either direct measurement of learning-induced changes in synaptic physiology using *in vivo* and *ex vivo* electrophysiological recordings (McKernan and Shinnick-Gallagher, 1997; Rogan et al., 1997) or through indirect measurements of saturation/occlusion or mimicry/manipulation of synaptic plasticity. For instance, cue-reward learning enhances AMPA receptor-mediated strength of thalamic synapses onto lateral amygdala neurons, which correlates with the level of learning in individual animals (Tye et al., 2008). In contrast, saturation of hippocampal synaptic strength prior to learning, by *in vivo* electrical stimulation-induced long-term potentiation (LTP), results in anterograde amnesia for acquisition of new spatial information, as well as the inability to adapt previously stored spatial relationships (McNaughton et al., 1986). Furthermore, mimicry experiments using optogenetic manipulation have proven that it is feasible to artificially modulate memory expression. For example, *in vivo* optogenetically-induced long-term synaptic depression (LTD) of auditory inputs in the lateral amygdala after learning impairs expression of auditory fear memory, which can be rescued by evoking LTP of these inputs (Nabavi et al., 2014). Additionally, pharmacological disruption of learning-induced and PKM-zeta mediated synaptic potentiation impairs memory storage and prevents successful memory retrieval (Pastalkova et al., 2006; Shema et al., 2011), although potential off-target effects of these manipulations demand further investigation. These and other studies provide evidence that learning-induced enhancement of synaptic connectivity is necessary for memory storage, however, they do not pinpoint whether these adaptations occur specifically in sparsely distributed engram cells or on a more global scale.

### Engram Cell-Specific Synaptic Adaptations Associated With Recent Memory

Converging evidence over the last few years indicates that learning-induced strengthening of synaptic connectivity occurs specifically between neurons that are activated at the time of learning. For instance, a CFC-induced increase in synapse density and AMPA/NMDA receptor current ratio has been reported in hippocampal dentate gyrus (DG) engram cells, which is prevented by post-learning inhibition of protein synthesis (Ryan et al., 2015). Furthermore, this study described enhanced connectivity between DG and CA3 engram cells compared to connectivity with neighboring non-engram cells in the CA3. Interestingly, in a mouse model of Alzheimer’s disease, contextual memory expression is impaired and learning does not increase spine density on DG engram cells, however, this spine and memory deficit can be rescued by optogenetically-induced LTP in these engram cells (Roy et al., 2017). At the synaptic level, pertinent proof for engram cell-specific enhancement in synaptic connectivity was provided by Choi et al. (2018), who developed dual-eGRASP to selectively label synapses between neurons activated by learning, their non-activated counterparts and the combinations thereof, with different fluorescent tags. They found an enhancement in synapse size and density on hippocampal CA1 engram cells that receive input from CA3 engram cells after fear conditioning, and showed that these adaptations supported memory strength and occluded subsequent LTP. At the molecular level, an early study (Matsuo et al., 2008) examined the dynamics of newly synthesized AMPA receptors by coupling the expression of GFP-GluA1 to activation of the *Fos* promoter. They found that new GluA1 subunits were selectively recruited to mushroom-type spines in hippocampal CA1 neurons 24 h after fear conditioning, suggestive of a learning-induced enhancement of AMPA receptor-mediated synaptic strength. Intriguingly, a follow-up study reported an overall reduction of spine density on learning-activated CA1 pyramidal neurons (Sanders et al., 2012), hinting toward a form of synaptic refinement through selective strengthening of a subset of spines on engram cells. Together, these studies provide strong evidence that learning induces an augmentation of (structural and physiological) synaptic connectivity between engram cells in the hippocampus and its necessity for the successful expression of recent memories.

In addition to the hippocampus, accumulating evidence points to adaptations in synaptic connectivity of engram cells in other brain regions. Lateral amygdala neurons tagged during auditory fear conditioning or discriminative fear learning exhibit enhanced pathway-specific synaptic strength with afferent neurons (Gouty-Colomer et al., 2016; Kim and Cho, 2017) and depotentiation of those same synapses causes memory loss (Kim and Cho, 2017). A very elegant study by Abdou et al. (2018) demonstrated that for memories that share a neuronal ensemble in the lateral amygdala, optogenetic potentiation and depotentiation of pathway-specific synapses selectively interfere with one memory while sparing the other. Motor skill learning has been shown to induce synaptic remodeling in a small subset of neurons in the motor cortex. These synapses were tracked with the expression of a photoactivatable Rac that targets activated synapses and the resulting memory can be disrupted by optical shrinkage of recently potentiated spines (Hayashi-Takagi et al., 2015). In the nucleus accumbens (NAc), an increase in silent synapses has been observed in activated neurons that mediate the expression of conditioned locomotor sensitization induced by cocaine treatment (Koya et al., 2012; Whitaker et al., 2016). Silent synapses contain functional NMDARs, but no functional AMPARs, and are considered to reflect an immature synaptic state (Rumpel et al., 1998). The functional relevance of the increase in silent synapses on activated neurons in the accumbens is unknown, but may reflect a transient state of these cells after the expression of the cocaine-evoked psychomotor sensitization which might be followed by recruitment of new AMPARs to these synapses. The latter has been observed at a global level in the NAc upon retrieval of remote cocaine memory (Wright et al., 2020) and in the dorsal hippocampal CA1 after retrieval of recent fear memory (Rao-Ruiz et al., 2011).

Together, these findings convincingly demonstrate that new learning is associated with enhanced synaptic connectivity involving increased pathway-specific potentiation of synapses between engram cells. Importantly, enhanced synaptic connectivity observed in the first days after learning is necessary for adequate recent memory expression, but whether it contributes to the persistence of an engram cell network is poorly understood.

### Global Synaptic Adaptations Associated With Remote Memory

Compared with adaptations that have been identified within the first week after conditioning, less is known about synaptic mechanisms that contribute to memory retention beyond this timeframe. At the structural level, an increase in spine density has been reported on neurons in the infralimbic and ACC after CFC (Restivo et al., 2009; Vetere et al., 2011a,b). In the ACC, the enhanced spine density is apparent 1 week after learning, then gradually increases over the following 5 weeks and lasts for at least 7 weeks (Vetere et al., 2011a). It is well-established that repeated exposure to psychostimulants, such as cocaine, also results in an increase in density of dendritic spines on cortical and striatal neurons and this is retained for at least 1 month (Robinson and Kolb, 1999). After motor learning, formation of new spines (5% of total spines) occurs on layer 5 pyramidal neurons in the primary motor cortex, as observed using *in vivo* two-photon imaging (Yang et al., 2009). Interestingly, the proportion of new spines that is maintained up to 2 weeks after motor learning is positively modulated by the number of training sessions and correlates with performance in this task. Yang et al. (2009) estimated that a small fraction of the newly formed spines (0.04% of total spines) is retained throughout the life of the animal and thereby represent a permanent trace of a learned experience in cortical networks. Of note, motor skill learning reflects a type of procedural learning, whereas most engram studies have thus far focused on associative learning and episodic memories. It is unknown whether the mechanisms of synaptic connectivity underlying these different forms of memory are fundamentally similar.

Several proteins have been discovered that contribute to memory persistence by altering structural connectivity. For instance, myocyte enhancer factor-2 (MEF2) is a transcription factor known to negatively regulate spinogenesis and viral-mediated enhanced expression of MEF2 in ACC pyramidal neurons during the first, but not seventh, week after fear conditioning prevents the learning-induced increase in spine density and subsequently impairs memory expression (Vetere et al., 2011a). The increase in spine density in the NAc after repeated cocaine exposure is also regulated by MEF2 (Pulipparacharuvil et al., 2008), but surprisingly, reducing spine density through enhancement of MEF2-mediated transcription promotes behavioral sensitization and cocaine conditioned place preference memory, suggesting that the cocaine-enhanced spine density serves as a compensatory mechanism to limit the effects of future cocaine exposure. This demonstrates that an experience-induced increase in spine density does not always underlie memory consolidation and should be interpreted with caution in the absence of causal evidence. Interestingly, MEF2 primes PSD-95 protein for degradation (Tsai et al., 2012) and disruption of PSD-95 function has been shown to impair retention of remote fear and ethanol conditioned place preference memory (Camp et al., 2011; Fitzgerald et al., 2015). With respect to fear memory, mice expressing a loss-of-function mutant PSD-95 show reduced neuronal activity in the infralimbic cortex during remote memory expression and decreased spine density and morphology in this region. Furthermore, CAMKIIα has a critical role in memory persistence, as mice with heterozygous CAMKIIα expression have impaired cortical LTP and show memory deficits at remote, but not recent, timepoints after learning (Frankland et al., 2001). Thus, several proteins involved in long-term structural plasticity of the synapse are critically contributing to memory persistence.

At the physiological level, evidence for long-lasting changes in synaptic function is predominantly obtained with appetitive learning paradigms. For instance, mice that receive repeated cocaine injections in an alternate context initially show synaptic depression (as measured by the AMPA to NMDA receptor current ratio) in neurons in the NAc, which reverses into synaptic potentiation at 10–14 days after the last cocaine treatment (Kourrich et al., 2007). Increased membrane expression of GluA1 and GluA3 subunits has been observed in the NAc at day 45, but not day 1, after cocaine self-administration, which is paralleled by enhanced presence of calcium permeable (i.e., GluA2-lacking) AMPA receptors (Conrad et al., 2008). In line with this, selective pharmacological blockade of calcium permeable AMPA receptors in the NAc attenuates time-dependent enhancement of conditioned cocaine seeking. Projections of the mPFC to the NAc core are thought to promote conditioned drug seeking, whereas projections to the shell may suppress drug seeking responses (Peters et al., 2009; Van den Oever et al., 2010). Cocaine self-administration initially generates silent synapses in both projections, but these are matured at day 45 of abstinence through recruitment of AMPA receptors (Ma et al., 2014). Interestingly, Ma et al. (2014) found that optogenetically-evoked LTD in the mPFC-NAc core projection attenuates conditioned cocaine seeking, whereas the opposite occurs after LTD-induction in the mPFC-NAc shell projection. Similarly, cocaine-induced locomotor sensitization is abolished after optogenetic reversal of synaptic potentiation in NAc D1-expressing medium spiny neurons (Pascoli et al., 2012). Regarding aversive conditioning, it was elegantly shown that transient loss of NMDA receptor subunit *GRIN1* expression in the forebrain abolishes LTP and when its expression is temporarily suppressed at 6 months after learning, this impairs the expression of a 9-month-old contextual and auditory fear memory (Cui et al., 2004).

Taken together, memory persistence is associated with enhanced synaptic connectivity through persistent changes in structural and physiological properties in brain regions that are engaged in a particular experience and prevention or reversal of these alterations disrupts memory retention. In the examples above, random neurons were selected and analyzed in relevant brain areas and therefore it remains to be determined whether the lasting adaptations described represent the engram in neurons that are activated during memory acquisition and retrieval.

### Engram Cell-Specific Synaptic Adaptations Associated With Remote Memory

Whereas fear memory-encoding engram cells in the hippocampal DG develop a rapid increase in spine density that diminishes within 2 weeks after learning, a more gradual increase in spine density occurs on engram cells in the mPFC which lasts for a least 2 weeks (Kitamura et al., 2017). Notably, selective blockade of synaptic transmission in DG engram cells prevents the subsequent increase in spines on mPFC engram cells, in line with the observations that post-learning synchronization and replay of hippocampal and cortical neuronal activity drives systems consolidation. However, whether the additional spines on mPFC engram cells represent enhanced synaptic connectivity with neurons in the same engram cell network or with other neurons is yet unknown. Using an elegant combination of genetic tagging (*Arc*-TRAP mouse line) with retrograde and anterograde tracing of activated neurons, Wall et al. (2019) found that neurons in the dorsal striatum that are activated by cocaine exposure receive monosynaptic input from widely distributed, but sparsely activated, cortical neurons. They show that synaptic strength (measured as postsynaptic AMPA receptor-mediated currents and presynaptic release probability) is selectively enhanced between cocaine-activated cortical and striatal neurons at 21 days after treatment. Interestingly, synaptic strength is further enhanced specifically between activated populations when animals are re-exposed to cocaine 15 days after repeated cocaine treatment and then persists for at least 4 more weeks. To date, these are, to our knowledge, the only studies that investigated synaptic connectivity of engram cells at a remote timepoint after a specific experience. Hence, the precise mechanisms that contribute to the stability of connectivity between engram cells and thereby support reactivation of an engram cell network during memory retrieval require further investigation.

## Memory Persistence Through Stable or Dynamic Adaptations in Synaptic Connectivity Between Engram Cells

**FIGURE 2:**
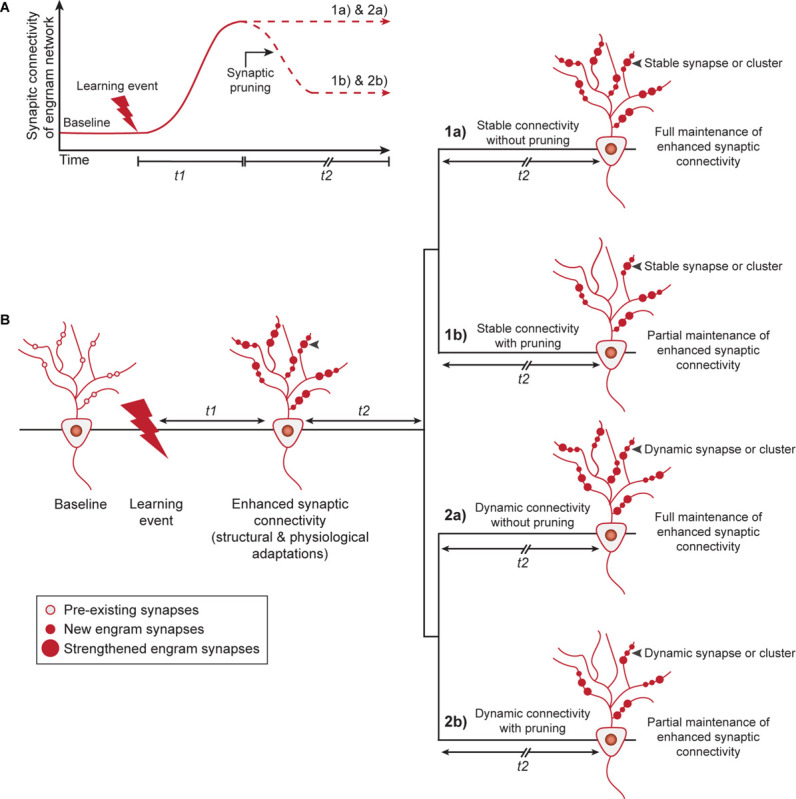
Memory persistence through stable or dynamic adaptations in synaptic connectivity between engram cells. **(A)** Learning induces structural and physiological adaptations of engram synapses (during t1) that results in enhanced synaptic connectivity within the engram cell network. After initial formation and strengthening of engram synapses, enhanced synaptic connectivity is fully maintained or pruning may lead to a partial retention of augmented connectivity within the engram cell network, with the purpose to promote the global memory encoding capacity of neural circuits. **(B)** Two potential models by which enhanced synaptic connectivity is fully or partially maintained to mediate memory persistence. Model 1 shows a stable state of engram synapse connectivity after t1 and predicts that synaptic alterations established by learning persist invariantly over time. This can occur either for all learning-induced synapses (1a) or for a subset of these synapses after synaptic pruning and refinement of the network (1b). Alternatively, Model 2 describes a dynamic view of synaptic connectivity and hypothesizes that the strength of individual synapses, possibly within a cluster, can be modified while keeping the overall connectivity of the engram cell network constant. This can either occur for all engram synapses/clusters (2a) or a subset of synapses/clusters after synaptic pruning and refinement of the network (2b). t1: days to weeks, t2: weeks to months. Gray arrowheads indicate an example of a synapse or synaptic cluster that is maintained stably (1a, 1b) or in a dynamic manner (2a, 2b) over time.

We will now discuss how enhanced synaptic connectivity, through structural and/or physiological adaptations (pre- and postsynaptic), could be maintained over time (Figure 2A). Unfortunately, at present, there is little experimental evidence to support a one-to-one correlation between memory retention and the individual synapses that contribute to the maintenance of this augmented connectivity. This would require long-term monitoring of the same population of engram cell synapses over a time-scale of weeks to months. Therefore, we will speculate about potential mechanisms and suggest one model that involves stable engram synapses that are defined at the time of learning and a second model of dynamic ongoing plasticity of synaptic connections between engram cells (Figure 2B). Furthermore, we complement both models with the possibility that synaptic pruning occurs in an engram cell network to preserve the global memory encoding capacity of neural circuits.

### Stable Synaptic Connectivity Without (1a) or With Pruning (1b)

In this model, enhanced learning-induced connectivity would endure immutably over the lifetime of a given memory due to unvarying size and strength of either (1a) all, or (1b) a subset of synaptic connections triggered by learning (Figure 2B). A handful of studies have, however, raised the possibility that spine dynamics may commensurate with the duration for which a memory is dependent on a particular population of engram cells. For instance, using two-photon microendoscopy, Attardo et al. (2015) found that dendritic spines in the hippocampus have a mean life-time comparable to the time-scale of systems level memory consolidation (approximately 15 days) and only transient increases in spine density were observed on DG engram cells after fear conditioning (Kitamura et al., 2017). Conversely, spine density on mPFC engram cells is enhanced remotely after learning (Kitamura et al., 2017) and at a global level, a small but significant fraction of new motor learning-induced cortical spines are retained over the lifetime of an animal (Yang et al., 2009). Thus, in line with their long-term contribution to memory persistence, neocortical dendritic spines also demonstrate heightened permanency relative to those in the hippocampus, supporting a role for stabilized patterns of synaptic connectivity within cortical networks and potentially between cortical engram cells and their subcortical targets.

The importance of activity-dependent synaptic pruning for the optimization of cortical network function and capacity during critical periods of development is well-established (Garey, 1984) and is also thought to have an important function during experience-dependent shaping of memory circuits (Holtmaat and Caroni, 2016). Therefore, we argue that it is likely that stable synaptic connectivity is maintained by only a subset of new and/or strengthened synapses after learning (Yang et al., 2009), i.e., scenario (1b), rather than by all engram synapses recruited during learning, i.e., scenario (1a). In support of this, pruning of motor learning-induced cortical spines occurs during rapid eye movement sleep, in addition to the strengthening of a subset of new spines (Li et al., 2017). Relatively weak engram synapses are most likely eliminated first and MEF2 may play an important role in this process (Caroni et al., 2014). Refinement of the engram cell network by gradual elimination of the majority, but not all, learning-induced new and/or strengthened synapses would enable some synaptic alterations to endure and maintain a partially augmented state of synaptic connectivity.

### Dynamic Synaptic Connectivity Without (2a) or With Pruning (2b)

An alternative view to a fixed synaptic connectivity configuration is a fluid model in which individual synapses on engram cells can be continually calibrated and reorganized (Routtenberg, 2013), while maintaining overall enhanced synaptic connectivity within the engram cell network. Computational studies lend credence to this model, wherein discrete synaptic weights within an engram cell network can fluctuate structurally and physiologically, while the overall connectivity strength of the memory circuit remains invariant (Abraham et al., 2019; Susman et al., 2019). Dynamic synaptic connectivity would enable a more malleable state of the network that would also account for the effects of homeostatic adjustments within the network, and assumes that a stored memory can outlive the strength of individual synapses that encode it. However, obtaining evidence for this dynamic model, at the level of both structural and physiological adaptations in the engram cell network, is technically challenging and would require the measurement of a population of synapses across extended times and different phases of behavior. Nonetheless, it is relevant to note that empirical evidence indicates that learning induces the clustered addition of dendritic spines and that this may serve to increase efficiency of information storage with relevant circuits of the brain (Fu et al., 2012; Frank et al., 2018). Although awaiting experimental evidence at the level of engram cells, in such a clustered plasticity model (Govindarajan et al., 2006), dynamic synaptic connectivity could be driven by bidirectional changes among individual synapses within a dendritic branch, while overall enhanced connectivity of the network remains unchanged. As with the stable synaptic connectivity model, we expect a dynamic model with synaptic pruning that results in a partially augmented engram cell network to have more physiological relevance (i.e., scenario 2b) than a model that assumes no spine turnover (i.e., scenario 2a). Dynamically clustered synaptic plasticity at a subset of engram-to-engram cell connections would enable efficient reactivation of the engram cell network upon memory retrieval (Govindarajan et al., 2006), while also limiting the use of resources to optimize memory capacity. Whether clustering, refinement and scaling of synaptic connectivity between engram cells takes place randomly or at specialized dendritic hotspots after learning is yet unknown.

Similar to structural connectivity, persistent physiological changes in synaptic strength can be supported by a stable or dynamic population of synapses between engram cells. So far, identified augmentation of synaptic strength has been measured by whole cell patch-clamp recordings, which do not provide insight into the specific synapses that are altered as a result of learning. Moreover, physiological adaptations likely also commensurate with the duration of engagement of engram cells in memory expression. For instance, DG engram cells exhibit enhanced synaptic strength the first days after fear conditioning (Ryan et al., 2015; Choi et al., 2018), but this may reflect a transient state similar to structural connectivity in the hippocampus. Experimentally-induced global LTP has been shown to persist for a considerable amount of time in the hippocampus (Abraham et al., 2002), arguing against a transient state of enhanced synaptic strength in hippocampal engram cells, however, it is questionable whether this represents a naturalistic mechanism. To date, strongest support for a persistent enhancement of pre- and postsynaptic properties has been observed between cocaine-activated cortical and striatal neurons several weeks after cocaine exposure (Wall et al., 2019). Future studies should address whether the alterations in synaptic strength are maintained by a stable or fluctuating pool of synapses over time-scales that reflect memory persistency.

The models we propose represent a dormant state of the memory after learning. Depending on epochs of retrieval and the conditions under which retrieval occurs, synaptic connectivity within the engram cell network may be altered, enabling updating of memory strength and content. This process is likely associated with modulation of physiological properties of existing synapses between engram cells (Wall et al., 2019), as well as addition/removal of synapses to reset connectivity within the network. Additionally, it may involve exclusion of already incorporated neurons or the engagement of new neurons in the engram cell network.

## Potential Molecular Mechanisms That Support the Persistence of Engram Synapses

**FIGURE 3:**
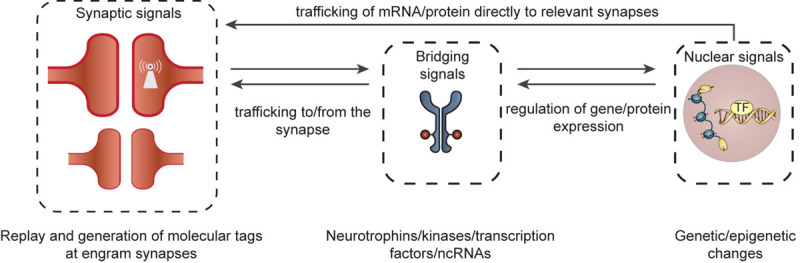
Organization of cellular feedback loops predicted to maintain augmented synaptic connectivity between engram cells. Ongoing molecular feedback between specific synapses and the nucleus would require coordinated communication between different cellular compartments in engram cells. This may be initiated by the formation of a learning-induced synaptic tag at relevant synapses (and/or clusters of synapses) within a dendritic branch. These molecular tags (e.g., self-perpetuating kinases, locally translated mRNAs, adhesion molecules, ion channels) may serve as synaptic signals that can, in turn, trigger self-sustaining neurobiological feedback loops, involving signal transduction molecules and bridging signals [e.g., kinases, neurotrophins, transcription factors (TF) or dendritic non-coding RNAs (ncRNAs)] between the synapse and nucleus. These bridging signals may then persistently regulate and direct transcriptional and translational programs via epigenetic (e.g., histone methylation/acetylation or DNA methylation) and genetic (e.g., transcription and growth factors) mechanisms within the nucleus/cytosol. The newly synthesized gene/protein products can then be relayed to discrete engram synapses/synaptic clusters enabling the modification or perpetuation of synaptic plasticity and a maintenance of overall synaptic connectivity of the engram cell network.

We will discuss a few of many possible mechanisms that can perpetuate synaptic connectivity within an engram cell network over weeks to months (for a more detailed review, see e.g., Smolen et al., 2019). Learning induces protein turnover (synthesis and degradation) that is necessary for remodeling of synaptic connectivity that drives memory formation and its stability over time. However, both gene and protein products have relatively short half-lives with proteins exhibiting an average turnover rate of 9 days in the brain (Price et al., 2010). Although experience can alter protein turnover of selected synaptic proteins (Heo et al., 2018), a majority of proteins known to constitute synapses are degraded and typically replaced within a time course of hours to days (Dorrbaum et al., 2018). Given this molecular turnover, how would the gene/protein machinery of enhanced synaptic connectivity be maintained over the time-course of memory persistence in either stable or dynamic synapses? Overall, it is likely that intracellular feedback occurs within and across specific cellular compartments, enabling signal transduction and regulation of discrete transcriptional/translational programs within engram cells. Here, we will focus our discussion on mechanisms involving persistent synaptic molecular tags that trigger synapse-to-nucleus biochemical signaling cascades and (epi)genetic adaptations (Figure 3). We propose that these mechanisms may work in concert to maintain engram cell connectivity patterns over the lifetime of a memory.

### Synapse-Specific Feedback Loops

The theory of synaptic tagging and capture (STC) (Frey and Morris, 1997) provides a mechanism by which connectivity between engram cells can be enhanced and sustained over time (Moncada et al., 2015). This would require the allocation of a persistent molecular tag or multiple tags to a subset of potentiated synapses within a dendritic branch/cluster on neurons that are activated at the time of learning and responsible for memory persistence. These ‘synaptic tags’ can subsequently trigger synapse-specific and self-sustaining molecular feedback loops, involving signal transduction, transcriptional, and translational pathways. In turn, information from the nucleus would travel only to individual, ‘tagged’ synapses or clusters of synapses (Govindarajan et al., 2006), as opposed to the remaining population of synapses on engram cells that are not involved in retention of a particular experience. This could maintain overall engram cell network connectivity via the regulation of synaptic gene programs, stimulation of local protein synthesis at the synapse and synapse-selective recruitment of glutamate and other receptors/channels to the postsynaptic density. Such mechanisms would have to take place recurrently (long after stimulation has ceased) during epochs of engram cell reactivation, when synaptic replay mimics activity patterns (intensity and temporal properties) that occur during initial memory formation (Ikegaya et al., 2004; Atherton et al., 2015; Wei et al., 2016; Giri et al., 2019; Smolen et al., 2019). Although attractive, this experience-specific and enduring STC-driven feedback remains largely speculative and the molecular tags remain to be identified. As discussed, transient forebrain-selective knock-out of neuronal *GRIN1* during the 7th month after learning, subsequently leads to a severe loss in expression of cued and contextual fear memory (Cui et al., 2004). This suggests that the amnesia is attributable to the disruption of NMDAR-dependent activation of downstream signaling pathways necessary to sustain adaptations underlying the maintenance of augmented synaptic strength. Additionally, learning-induced, and AMPA and NMDA receptor-mediated, recruitment of cortical neurons in remote memory is paralleled by, and dependent on, epigenetic modifications in the cortex (Lesburgueres et al., 2011). This potentially drives transcriptional programs to support remote memory retention. Together, these studies provide preliminary evidence for synapse-driven feedback loops in memory persistence, however, these theories require more experimental evidence at an engram cell-specific level, and over time-scales relevant to the persistence of remote memories. Although the identity of spatially restricted synaptic tags is largely unknown, there are several potential candidates. Synaptic tags may involve long-lasting proteins whose turnover is altered upon learning, mRNAs that are locally transcribed at the synapse, adhesion molecules, ion channels and self-perpetuating “cognitive kinases” (Martin and Kosik, 2002; Sajikumar et al., 2005; Heo et al., 2018; Smolen et al., 2019). Next, feedback would require coordinated and ongoing communication between different cellular compartments, such as between specific synapses and the nucleus. These signal transduction pathways or ‘bridging signals’, although unidentified for engram cells, may involve several synaptonuclear signaling proteins including kinases, neurotrophins, transcription factors or dendritic non-coding RNAs [reviewed in detail elsewhere: (Thompson et al., 2004; Ch’ng and Martin, 2011; Karpova et al., 2012; Herbst and Martin, 2017)].

### Genetic Feedback Loops

Regulated transcriptional/translational programs within engram cells that are ongoing long after learning/stimulation occurs are considered to contribute to a lasting increase in synaptic connectivity. Indeed, transcriptome profiling of mPFC cells activated by cocaine exposure (Ye et al., 2016) or fear conditioning (Ye et al., 2016; Chen M. B. et al., 2020) demonstrated that these cells express newly synthesized molecular signatures weeks (14–16 days) after the experience, including the expression of genes involved in presynaptic function (Chen M. B. et al., 2020). Transcription and growth factors likely participate in transcriptional feedback loops initiating and perpetuating gene expression programs that underlie engram cell stability and memory storage. The transcription factor CREB, a well-studied example, is crucial for mechanisms of intrinsic and synaptic plasticity (Barco et al., 2002), and thereby regulates allocation (Dong et al., 2006; de Armentia et al., 2007; Zhou et al., 2009; Yiu et al., 2014), consolidation (Bourtchuladze et al., 1994) and linking of memory (Cai et al., 2016) across several brain regions and memory types. We recently demonstrated that CREB-mediated transcription in DG (Rao-Ruiz et al., 2019) and mPFC (Matos et al., 2019) engram cells is necessary for the consolidation of recent and remote contextual fear memory, respectively. However, whether and how sustained CREB-mediated transcription in the nucleus leads to the maintenance of augmented synaptic connectivity patterns over time is currently unknown. Although direct experimental evidence is lacking, feedback loops initiated at the engram synapse may result in auto-activation of CREB-mediated transcription via CRE elements in the promoter region of CREB (Meyer et al., 1993). In addition to the regulation of gene products that can be targeted to specific synapses, CREB-mediated elevation of intrinsic neuronal excitability may increase the efficiency of relevant synaptic connections within the engram cell network during synaptic replay events (Lisman et al., 2018). In addition to CREB, growth factors can also participate in the maintenance of engram cells, either alone or in a reciprocal interaction with CREB. For example, the growth factor BDNF has emerged as an important regulator of neuronal structure, functional connectivity, synaptic plasticity and learning (Schinder and Poo, 2000; Tyler et al., 2002; Bekinschtein et al., 2008). Along with CREB, global hippocampal BDNF levels can persist for at least 20 h after learning (Bambah-Mukku et al., 2014), and delayed autoregulatory BDNF expression, 12 h after Inhibitory Avoidance (IA) learning, has been shown to be critical to the persistence of IA memory for at least 7 days (Bekinschtein et al., 2007). In DG engram cells, activation of CREB-mediated transcription and upregulation of BDNF is apparent at 24 h after contextual fear conditioning (Rao-Ruiz et al., 2019). Thus, although lacking direct experimental evidence over periods of weeks to months, it is possible that transcriptional feedback loops involving CREB, BDNF or other transcription and growth factors participate in the maintenance of synaptic connectivity patterns over weeks, months or even years.

### Epigenetic Feedback Loops

As described above, synaptic triggers can lead to epigenetic modifications that regulate transcriptional signatures supporting remote memory. In line with this, learning-induced chromatin modifications (e.g., histone methylation/acetylation or DNA methylation) have been measured at a global level in the hippocampus and prefrontal cortex (Miller et al., 2010; Graff et al., 2014; Zovkic et al., 2014; Halder et al., 2016), and recently in an engram cell-specific manner in the hippocampal DG (Gulmez Karaca et al., 2020; Marco et al., 2020). These studies demonstrate that such epigenetic adaptations are important for the consolidation, retrieval, updating and maintenance of memories. For instance, changes in methylation state of synaptic plasticity genes in cortical neurons have been observed long (4 weeks) after contextual fear conditioning (Halder et al., 2016). Moreover, these changes displayed a significant overlap with chromatin modifications observed 1 h after conditioning (Halder et al., 2016). Thus, epigenetic mechanisms may play a pivotal role in self-sustaining feedback loops regulating the expression of persistent transcriptional signatures that (1) are necessary for the retention of enhanced synaptic connectivity within the engram cell network, or (2) could prevent engram cell connectivity patters from being overwritten in the face of new experience-driven plasticity (Kyrke-Smith and Williams, 2018). However, how epigenetic feedback loops could support the maintenance of specific engram synapses remains to be investigated.

## Conclusion and Future Perspectives

Accumulating evidence indicates that expression of remote memory depends on cortical neurons that are activated at the time of memory acquisition. After learning, consolidation is thought to enhance synaptic connectivity within the engram cell network through structural and physiological adaptations, thereby supporting memory retention. Based on this, we propose that the connectivity patterns that support persistence of an engram cell network can be perpetuated over time in different ways. Whether augmented connectivity is indeed maintained by stable or dynamic synapses and whether it involves synaptic pruning is an important topic for future research. To date, experimental evidence that provides detailed insight into the mechanisms that contribute to the persistence of synaptic connectivity between engram cells, such as the role of synapse turnover, and the identity of molecular mechanisms that coordinate and sustain these processes, is limited. A handful of interconnected synaptic and (epi)genetic feedback loops have been identified for specific types of memory at a global level of analysis. However, the picture remains incomplete, and the palette of recursive mechanisms that direct plasticity to specific sites and connections within the engram cell network long after learning occurs, remains largely unknown. Furthermore, even though we have focused mainly on mechanisms of enhanced synaptic connectivity underlying memory retention, non-synaptic mechanisms [e.g., persistent changes in excitability, (epi)genetic adaptations that are not connectivity related] may also play a role and need to be further investigated in order to obtain a comprehensive picture of the cellular and molecular processes that support memory persistence.

The field of learning and memory is currently in a fortunate position, with a highly specialized and rapidly developing technical toolbox; including, but not limited to, *in vivo* imaging techniques to measure brain circuit dynamics, permanent cell/synapse-specific tagging methods, ultrasensitive -omics techniques, and spatio-temporally precise manipulation tools (Sakaguchi and Hayashi, 2012; Chen et al., 2013; Josselyn et al., 2015; Yang and Yuste, 2017; Choi et al., 2020). Although challenging, this repertoire of techniques positions the field with unprecedented opportunities to dissect the complex architecture of memory persistence across multiple levels of analysis. A first step toward delineating how enhanced connectivity patterns are stabilized within an engram cell network would involve chronic, longitudinal *in vivo* imaging of engram cell synapse dynamics over time-scales that reflect the persistency of remote memories. This might also reveal whether enhanced connectivity occurs in functional clusters exclusive to synapses between engram cells of interconnected brain regions, or whether this is maintained by engram synapses that function as single units. Next, the identification of synaptic tags, signaling mechanisms, epigenetic modifications and transcriptional/translational events specific to engram cells would require temporal molecular analysis of these neurons and the specific synapses between them. This could be addressed by combining permanent fluorescent tagging of engram cells/synapses with the generation of molecular profiles of these compartments. Finally, causal manipulation of identified molecular adaptations specifically in engram cells is crucial to elucidate how changes that maintain enhanced synaptic connectivity support the stabilization of an engram cell network over time. It is noteworthy that thus far, the majority of studies focused on Fos- or Arc-expressing engram cells. Fos- and Arc-expressing neurons overlap to a large extent after learning (Rao-Ruiz et al., 2019), but evidence suggests that Fos-expressing neurons represent a subpopulation within a larger engram cell network (Sun et al., 2020). Distinct engram cell subpopulations may potentially maintain enhanced synaptic connectivity through different mechanisms and/or underlying molecular feedback loops. In addition to alterations that occur within the engram cells, research focusing on the potential role of neuromodulation, inhibitory microcircuits and non-neuronal cell types in the maintenance of stability of a memory engram is highly relevant. In conclusion, elucidating the molecular, structural and physiological framework of engram synapse-specific mechanisms is imperative to decode how synaptic connectivity within an engram cell network can be strengthened, maintained and updated to support the longevity of a memory.

## Author Contributions

All authors listed have made a substantial, direct and intellectual contribution to the work, and approved it for publication.

## Conflict of Interest

The authors declare that the research was conducted in the absence of any commercial or financial relationships that could be construed as a potential conflict of interest.
